# Endometrial delay is found to be part of a normal individual dynamic transformation process

**DOI:** 10.1007/s00404-021-06086-8

**Published:** 2021-05-19

**Authors:** Joachim Alfer, Roxana M. Popovici, Amir Fattahi, Jürgen Krieg, Ralf Dittrich, Matthias W. Beckmann, Arndt Hartmann, Nathalie Bleisinger

**Affiliations:** 1 Department of Pathology, Erlangen University Hospital, Friedrich–Alexander University of Erlangen–Nürnberg, Erlangen, Germany; 2 Kaufbeuren–Ravensburg Institute of Pathology, Elisabethenstrasse 19, 88212 Ravensburg, Germany; 3 Munich Fertility Center, Munich, Germany; 4 Women’s Reproductive Health Research Center, Tabriz University of Medical Sciences, Tabriz, Iran; 5 Department of Obstetrics and Gynecology, Erlangen University Hospital, Friedrich–Alexander University of Erlangen–Nürnberg, Erlangen, Germany; 6 Amberg Fertility Center, Amberg, Germany

**Keywords:** Endometrium, Maturation, Delay, Receptivity

## Abstract

**Purpose:**

Limited information is clinically available concerning endometrial receptivity; assessing endometrial transformation status is therefore an urgent topic in assisted reproductive technology. This study aimed to investigate individual endometrial transformation rates during the secretory phase in subfertile patients using personal endometrial transformation analysis.

**Methods:**

Monitoring was carried out during the secretory phase to obtain endometrial receptivity profiles. For the investigation, two endometrial biopsies were taken within one menstrual cycle. The extended endometrial dating was based on the Noyes criteria, combined with immunohistochemical analyses of hormone receptors and proliferation marker Ki-67. Biopsies were taken mainly at days ovulation (OV, *n* = 76)/hormone replacement therapy (HRT, *n* = 58) + 5 and + 10.

**Results:**

The results of the two biopsies were correlated with the clinically expected day of the cycle and showed temporal delays or hypercompensations, diverging from the expected cycle days by 0.5–5 days. In comparison with the first biopsies, the transformation rate in the second biopsies showed compensation, augmented delay, or constant transformation in 48.69, 22.37, and 28.94% of cases for ovulation in natural cycles and 56.89, 25.85, and 17.26% for HRT cycles, respectively.

**Conclusion:**

The study revealed an individually dynamic transformation process of the endometrium, with the ability to compensate or enlarge an initial “delay”, which is now identified as a normal individual transformation process during the secretory phase. This information is of great importance for the scientific investigation of dynamic changes in endometrial tissue, as well as for the timing of embryo transfers.

## Introduction

Up to 10% of couples who are unable to achieve pregnancy after a year of frequent, unprotected intercourse are defined as subfertile [1]. Although in vitro fertilization/intracytoplasmic sperm injection (IVF/ICSI) may be able to help these patients, live birth rates in even the most successful fertility treatment centers are only 30% per cycle [2].

Repeated implantation failure (RIF) is defined as a minimum of three unsuccessful transfers of one or two high-quality embryos [3, 4]. The underlying reason for RIF remains unknown; in most cases, the transferred embryos are of optimal quality and the endometrium shows a triple-line pattern with a thickness of more than 6 mm. It is thought that a lack of adequate endometrial receptivity is responsible for up to two-thirds of unsuccessful embryo transfers [5]. The relevance for implantation of many so-called endometrial receptivity biomarkers, such as cytokines [6–9] and receptors [10, 11] has been investigated in the recent years. These markers can be detected in the endometrium during the window of implantation [12–19].

The process of secretory transformation of the endometrium into the receptive stage was thought to be well known. A triple-line pattern in the endometrium that is detected on ultrasound during the follicular phase and a minimum endometrial thickness of 7 mm are signs of good endometrial quality [20]. Nevertheless, pregnancies do also occur when the endometrium has a thickness of less than 7 mm [21, 22]. The development of the endometrium after ovulation has been thought to follow a fixed transformation sequence. In the standard protocol, the time for embryo transfer is 5 days after ovulation or progesterone administration. The quality of the secretory transformation process in the endometrium cannot be assessed using clinical examinations, such as vaginal ultrasound.

The endometrium is responsible for controlled implantation processes, and it can be postulated that it is the main factor involved in the high rate of implantation failure (up to 66%), even when the embryo is in good condition [5]. Endometrial quality has been investigated in several studies, which have detected one or more molecular markers for endometrial receptivity [6, 9, 15, 17, 23–26]. Unfortunately, most of the studies on endometrial receptivity and biomarkers for it have investigated endometrial biopsies without carrying out histological dating, or detecting hormone receptors or proliferation marker Ki-67 [27–29]. Histomorphological dating is possible using the criteria defined by Noyes et al. (originally published in 1950 in *Fertility and Sterility*), which are regarded as the gold standard for endometrial dating [30].

As it is thought that subfertile patients may have a problem involving endometrial receptivity, the aim of this study was to monitor individual endometrial transformation rates during the secretory phase at the day of embryo transfer (ovulation (OV)/progesterone (P) + 5) and at the expected end of the implantation window (OV/P + 10) within one menstrual cycle. This analysis is combined with an extended endometrial dating method [31] that generates an endometrial receptivity profile. The intention was to verify whether the expected histological days that define the opening and closing of the implantation window are reached or undergo any sort of arrest or delay during the secretory phase. It was expected that new information would be obtained regarding dynamic endometrial transformation and thus evidence capable of clarifying recurrent implantation failure in patients. The new insights obtained may provide valuable information for the optimal timing of embryo transfers.

## Materials and methods

### Study population and biopsy sampling

The study investigated 76 patients (mean age 37.72 ± 3.8 years) with well-monitored natural mock cycles and 58 patients (mean age 36.5 ± 4.6 years) receiving a hormone replacement therapy (HRT) in mock cycles. A total of 152 endometrial biopsies were mainly obtained on ovulation (OV) days OV + 5 and OV + 10 from individuals in the natural cycle, and 116 biopsies were collected on progesterone (P) days P + 5 and P + 10 from patients in the HRT cycle, corresponding to days 19 and 24 of the menstrual cycle. A Pipelle endometrial suction curette (Gynemed GmbH, Lensahn, Germany) was used for sampling. For the current transformation analysis, endometrial biopsies were taken at OV/P + 5 and OV/P + 10; also, depending on individual clinical management, between OV + 4 and OV + 11. The biopsies were to be a minimum of 5 mm and a maximum of 10 mm in size. Biopsies were taken from different areas—for example, once from the posterior wall and once from the anterior wall of the uterine cavity—to avoid taking biopsies twice from the same area within the interval of 5 days. HRT was administered on the basis of the standard Kaufmann protocol [32], including estrogen and also progesterone administration. The patients’ clinical investigations did not reveal any pathological results, and they had adequate ovulation, optimal hormone levels in the peripheral blood, and typical transformation of the endometrium with a triple-line pattern as detected on ultrasound. Patients with abnormal uterine cavities on sonohysterography, unilateral, or bilateral hydrosalpinx, and endometrium ≤ 6 mm were excluded from the study. The approval for the study was obtained from the ethics committee of the Medical Faculty of Friedrich–Alexander University of Erlangen–Nürnberg.

### Endometrial dating method

The method used assigned the modified dating analysis, with expression patterns of the hormone receptors and Ki-67, to each histomorphological appearance on cycle days 16–24 [31]. The endometrial biopsies were analyzed in accordance with previously published criteria [15, 33]. Various parameters were taken into consideration to perform endometrial dating, including menstrual cycle days, histological dating criteria based on the Noyes protocol [30], and the immunohistochemical expression pattern of the estrogen receptor, progesterone receptor, and proliferation marker Ki-67 in all 268 biopsies.

The receptor analyses focused on glandular epithelium in the stratum functionale of the complete endometrium biopsy. The biopsies were analyzed and classified at interval steps of 5%. Counting was also done for results with less than 5% of stained nuclei. Ki-67 analysis was also done on the glands of the stratum functionale. Stromal cells were analyzed by counting 300 stromal cells in three representative fields.

### Immunohistochemistry of endometrial markers

The endometrial biopsies were fixed and stained as described previously [17, 31]. Briefly, the slides were incubated with diluted primary monoclonal antibodies (1:300, 1:400, and 1:500 for progesterone receptor, estrogen receptor, and Ki-67, respectively) for 45 min at room temperature (Table 1).

**Table 1:** Antibodies used

Monoclonal (mouse)			
Progesterone Receptor	PR	1: 300	DCS, Hamburg, Germany
Estrogen receptor	ER	1: 400	DCS, Hamburg, Germany
MIB-1	Ki-67	1: 500	Zytomed, Hamburg, Germany

After the slides had been washed in a washing buffer (Zytomed Systems GmbH, Berlin, Germany) and incubated with PostBlock reagent (POLAP-100 Kit, Zytomed), they were incubated with alkaline phosphatase polymer (POLAP-100 Kit, Zytomed) for 30 min at room temperature. To confirm the immunostaining method, positive control tissues were attached to each slide. For Ki-67, an appendix cross-section served as the positive control and a cell control array was used for hormone receptors (Zytomed, cat. no. MB-CC REZ).

## Results

**Fig. 1:**
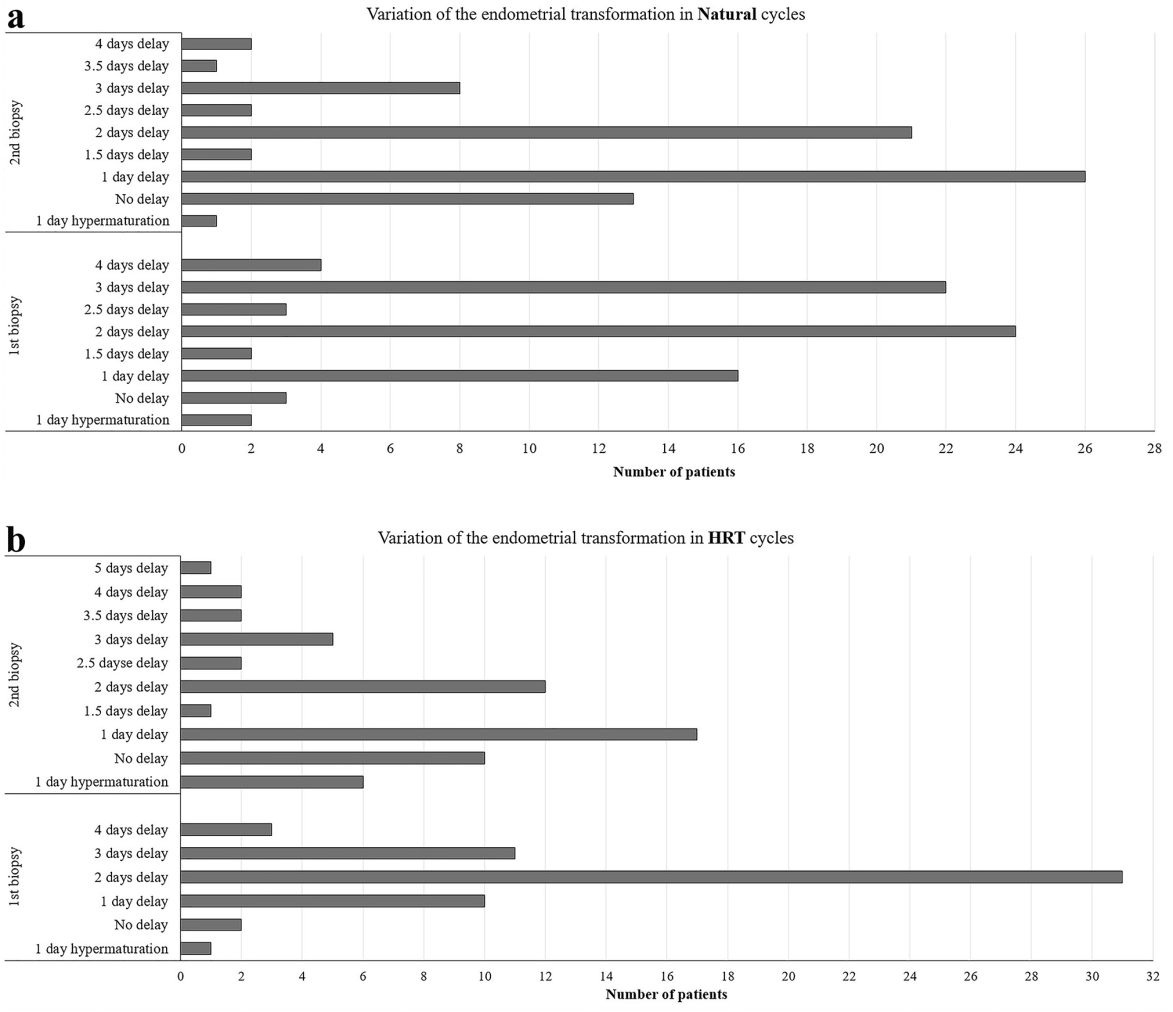
Variation of endometrial transformation in natural and HRT cycles. Endometrial transformation on days OV/P + 5 and + 10 of 76 natural cycles and 58 hormone replacement therapy (HRT) cycles. **a** Variation in endometrial transformation among natural cycles. **b** Variation in endometrial transformation among HRT cycles

The two biopsies taken showed variable dynamic secretory changes with delayed, constant, and accelerated development (Fig. 1a, b), but mostly delayed endometrial transformation and individually differing progression to the expected day of the cycle; however, accelerated transformation was seen in three cases in the first biopsy and seven cases in the second biopsy. Biopsies taken at OV + 5/P + 5 and OV + 10/P + 10 showed a mean test result corresponding to a cycle day of OV + 2.95 ± 1.17/P + 3.04 ± 0.93 and OV + 8.6 ± 0.97/ P + 8.65 ± 1.38, respectively. The mean delays in the first and second biopsies in the natural and HRT cycles were 2.05 ± 1.39 and 2.0 ± 0.94 days for first biopsies, and 1.45 ± 0.97 and 1.32 ± 1.37 days for second biopsies, respectively. Interestingly, in the natural cycle biopsies, only three and thirteen of the cases were at the expected transformation dates in the first and second biopsies, respectively (Fig. 1a). In the HRT cycle, two and ten cases were observed with timely transformation at the first and second biopsies, respectively (Fig. 1b).

**Fig. 2:**
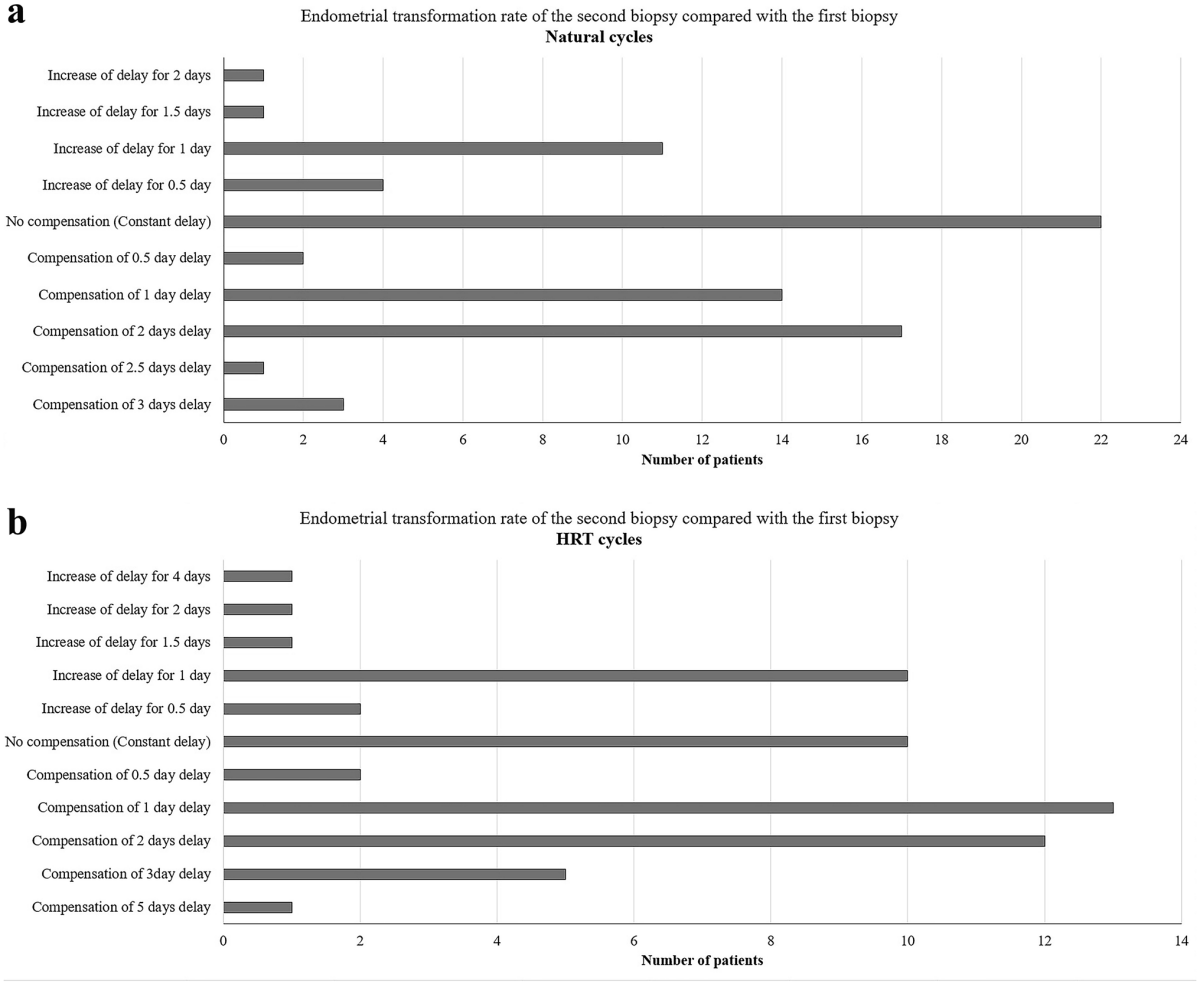
Comparison of endometrial transformation rates in natural and HRT cycles. Endometrial transformation on days OV/P + 5 and + 10 of 76 natural cycles and 58 hormone replacement therapy (HRT) cycles. **a** Comparison of transformation in the second biopsies (OV + 10) with the first biopsies (OV + 5) in the patients with natural cycles. **b** Comparison of transformation in the second biopsies (P + 10) with the first biopsies (P + 5) in patients with HRT cycles. *O*, ovulation in natural cycles, *P* progesterone administration in HRT cycles

**Fig. 3:**
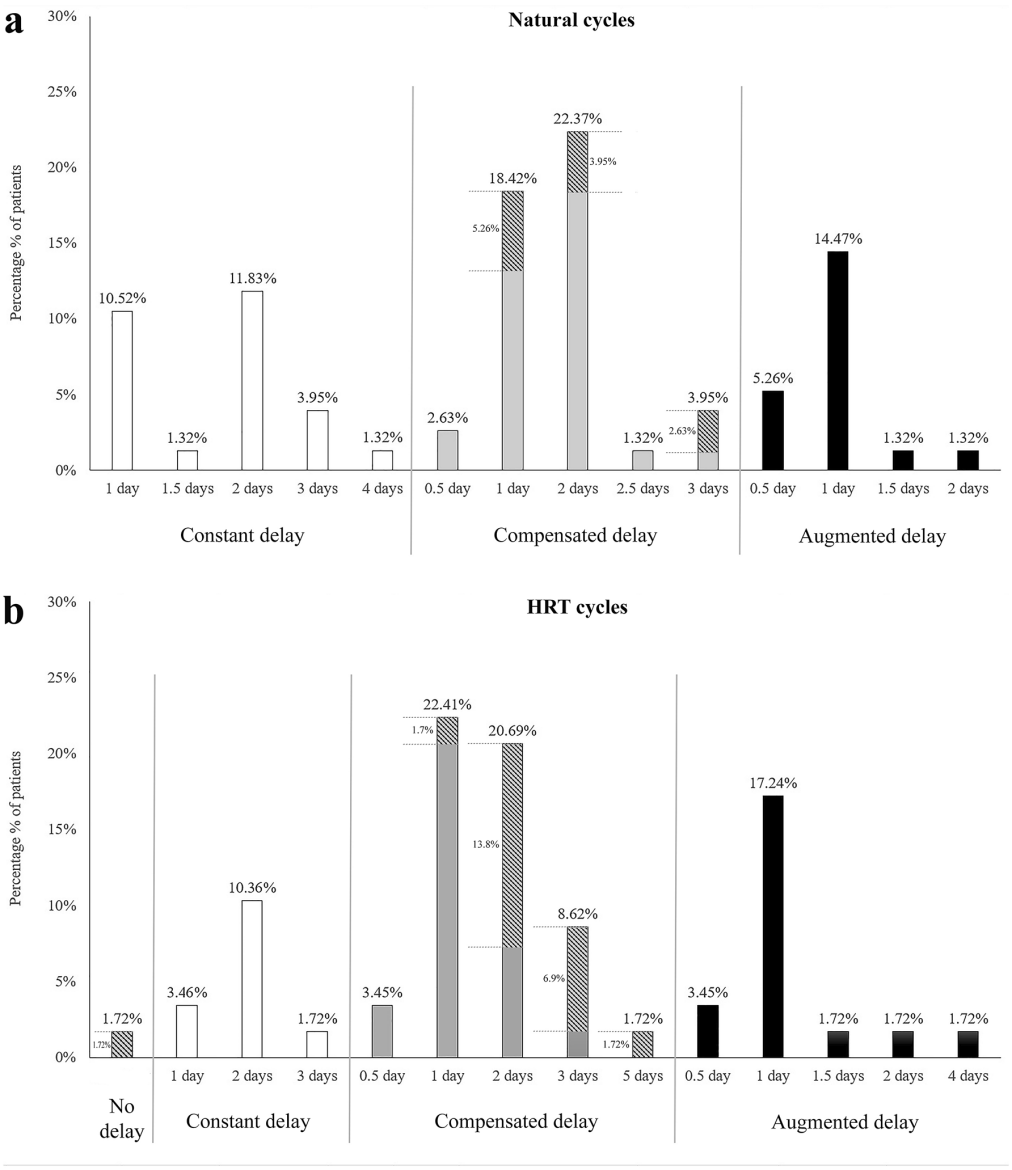
Behavior of endometrial transformation speed between first and second biopsies of natural and HRT cycles. Constant, compensated, and augmented delay in the endometrial tissues observed on day OV/P + 10 in comparison with day OV/P + 5. **a** Constant, compensated, and augmented delay in the natural cycles; **b** constant, compensated, and augmented delay in the HRT cycles. The hatched parts of the columns in compensated delays in **a** and **b** indicate the percentage of endometrium that was able to enter the expected day of transformation (OV/P + 10) or hypercompensation (OV/P + 11). *OV* ovulation in natural cycles, *P* progesterone administration in hormone replacement therapy (HRT) cycles

The transformation rate between two samples (OV/P + 5 to + 10) showed constant delay, compensation for delay or augmented delay in 28.94, 48.69, and 22.37% of cases in the natural cycles and 17.26, 56.89, and 25.85% of the HRT cycles, respectively (Fig. 2a, b). The correspondence with the expected transformation day (OV/P + 10) was only observed among the endometrial samples in which there was compensated transformation. The percentages of samples that reached the expected transformation day were 11.84% and 25.84% for natural and HRT cycles, respectively (Fig. 3a, b).

**Fig. 4:**
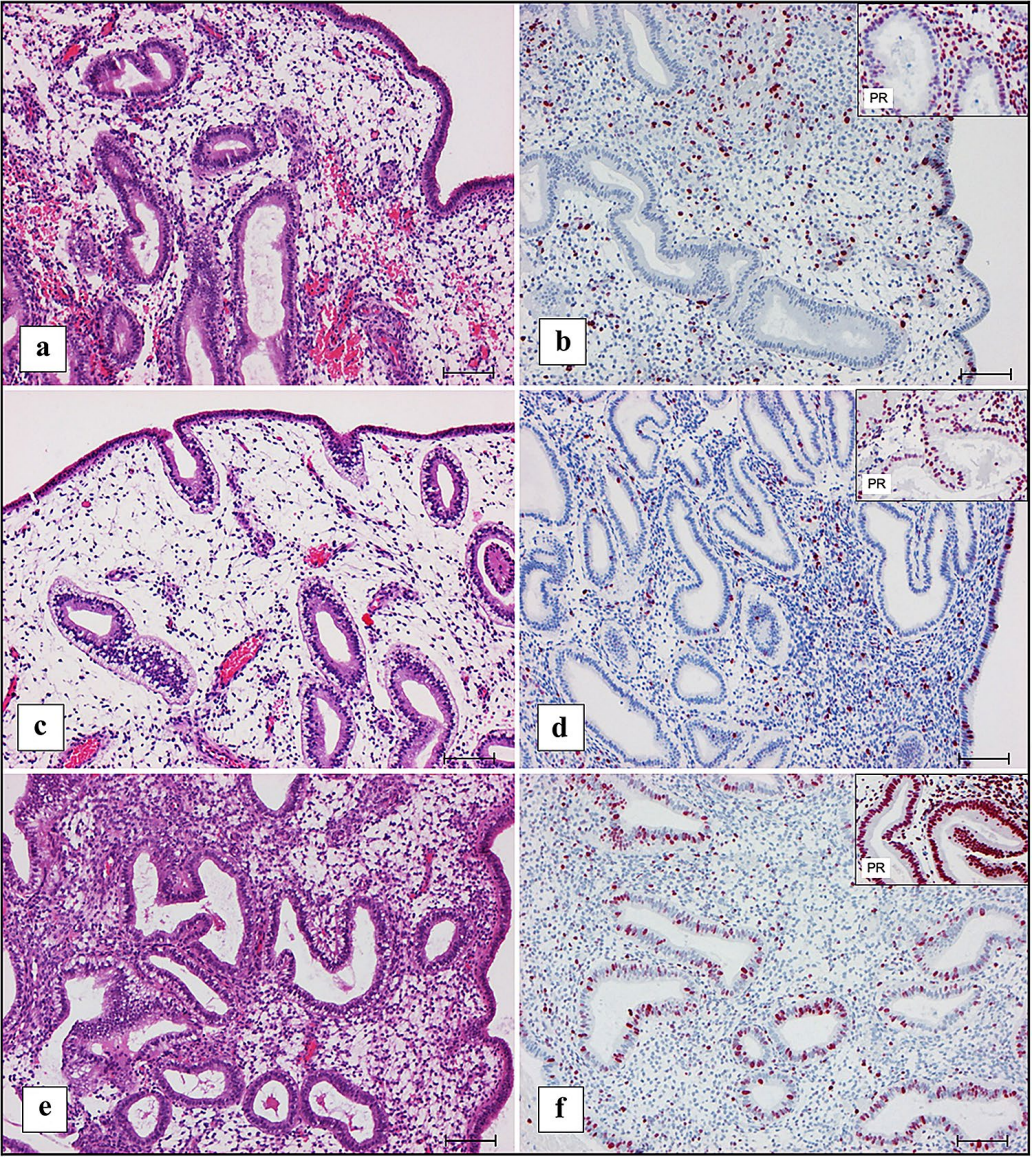
Comparison of histomorphological and immunohistochemical pattern between cycle day 19, day 17, and day 16. Original magnification 100 × , bar 100 µm in all figures and inserts. **a** Hematoxylin–eosin (HE) staining, histomorphological day 19 (clinically expected day at OV/P + 5) with scattered small, retronuclear glycogen vacuoles of glandular epithelium. **b** Immunohistochemical detection of Ki-67. Nuclei of glandular epithelium are negatively and nuclei of stromal cells are positively stained in a higher percentage in comparison with (**d**) and (**f**). Insert: progesterone receptor (PR), nuclei of glands positive in less than 50%, stromal cells positive in about 90%. **c** HE staining, histomorphological day 17 (mean result at clinical day OV/P + 5) with continuously visible conspicuous retronuclear glycogen vacuoles of glandular epithelium. **d** Immunohistochemical detection of Ki-67: nuclei of glandular epithelium are positively stained in a few cells and low in stromal cells. Insert: PR, nuclei of glandular epithelium are positive in more than 50% of cells, stromal cells positive in about 90%. **e** HE staining, histomorphological days 15–16 (expected at clinical day OV/P + 1–2), with scattered small, retronuclear glycogen vacuoles of glandular epithelium. **f** Immunohistochemical detection of Ki-67: nuclei of glandular epithelium are positively stained in about 25% of cells and in about 5% of stromal cells. Insert: PR, nuclei of glandular epithelium are positive in more than 90% of cells, stromal cells positive in about 90%

The expected histomorphological and immunohistochemical results for the first biopsy taken at OV/P + 5, which correlates to the cycle day 19, are shown in Fig. 4a and b. The mean results of first biopsies indicated a reduced transformation, which correlated with cycle day 17 (OV/P + 3) (Fig. 4c, d). However, transformations correlating with cycle day 16 (OV/P + 2) and earlier were also detected (Fig. 4e, f). For the first biopsies, a reduced transformation of more than 2 days was seen in 38.16 and 24.14% of the OV and HRT cycles, respectively.

The fastest transformation for natural cycles between the first and second biopsies was seen in three patients, representing a transformation speed of eight histomorphological days within 5 days of cycle. In HRT cycles, fast transformation was detected in five patients. In one patient, the endometrium at the first biopsy (P + 5) correlated with P + 1 and after five cycle days it reached day P + 11. Therefore, the endometrium transformed by 10 histomorphological days within 5 days of cycle. The slowest maturation was detected during one HRT cycle. This patient almost had arrested maturation, developing only 1 day of histological transformation after five cycle days (day P + 4 to P + 5).

Reactive changes due to the first biopsy, such as fibrous changes or enhanced vascularization—which would be normally seen after biopsy in other tissues such as skin—were never observed in the second biopsies from the endometrium.

Active endometritis was seen in a second biopsy from one patient with active endocervicitis, detected retrospectively in the first biopsy.

Negative controls resulted in no staining and positive controls reacted as expected.

## Discussion

To the best of our knowledge, this is the first study in which individually differing dynamic endometrial transformation rates have been observed within the secretory phase in natural and HRT cycles by monitoring the human endometrium. The results clarify one aspect of endometrial factors as a cause of subfertility. The coordination of timing between an embryo with implantation potential and a receptive endometrium appears to be a key factor in increasing the chances of pregnancy. In a natural pregnancy, coordinating the timing is a physiological process, as the embryo develops into the mother’s reproductive tract and so can be synchronized with the endometrium. The detected variation in the endometrial transformation process to enter the receptive window of implantation will therefore be the same for the embryo in vivo. There is possibly an individual, constantly adjusted receptivity pattern for each woman in each menstrual cycle. It can be postulated that endometrial-related infertility may occur when the synchronization process is interrupted and the endometrium and the embryo are at different receptive stages. Nevertheless, these cases were able to enter the window of implantation. In OV cycles, one might suggest a kind of corpus luteum insufficiency as a reason for the low onset of maturation, but since progesterone administration in HRT cycles yielded the same delay, it can be concluded that the delay was the result of a disturbed progesterone-related regulatory process that may lead to an abnormal synchronization process between the embryo and the endometrium. In IVF/ICSI procedures, however, the embryo develops in the culture medium, so that synchronization needs to be taken into consideration through timing of the embryo transfer. The receptive stage of the endometrium and the correct time for embryo transfer; therefore, need to be determined in each woman after implantation failure of high-quality embryos in an IVF/ICSI cycle. These patients might have a deviation of the expected transformation day of cycle, or other reasons like endometritis.

The new method of analysis presented here involves a combination of histological and immunohistochemical investigations and provides an endometrial receptivity profile. The results show that the speed of dynamic transformation of endometrial tissue differs individually, usually with a mean delay of approximately 2 days in the early secretory phase. In addition, the second biopsy at the end of the window of implantation revealed that the secretory changes are individually dynamic, as it detected constant delay, ongoing retarded transformation, or compensation for delay. These results provide new insights into the behavior of the endometrium, which does not develop day by day during a constant period of 14 secretory days as suggested by Noyes and colleagues.

It should be mentioned that it may be difficult and confusing to distinguish between cycle days 19 and 16 using the histomorphological method, but it is very important, since marker molecules for endometrial receptivity are already visible at day 19, but never at day 16. This information is also relevant for decision making on embryo transfers. However, using the current immunohistochemical analysis led to unambiguous assignment of the endometrial transformation on these days, because cycle day 19 shows a marked reduction in hormone receptors and Ki-67 in the glandular epithelium, whereas the receptors and Ki-67 are strongly expressed in the glandular epithelium on day 16 and earlier (Fig. 4). When the results of second biopsies were compared on the same cycle day with single biopsies in our recent article [31], no differences in the receptor expression pattern were detected.

In earlier studies using the Noyes criteria, endometrial biopsies with more than 2 days of difference between the histomorphological dating and the expected day after ovulation were considered to be “out of phase” [34, 35]. On this basis, out-of-phase endometrium has been reported in 5–50% of patients [36–38]. Murray et al. also reported that up to 26% of endometrial biopsies from fertile volunteers taken 6–10 days after ovulation showed a delay of 2 days or more [39]. This has led to the view that the Noyes criteria are not accurate.

Beyond morphological dating of the endometrium, several studies have additionally focused on one or more molecular markers in order to improve the use of histopathological dating. Analysis of integrin expression in parallel with histomorphological dating has been carried out, for example, but the method also appeared to be inadequate for predicting endometrial receptivity [23, 40, 41]. This might be the result of inadequate histological dating in comparison with the present extended dating method. Research studies have also investigated the potential for a molecular classification of the endometrium using transcriptomic profiling [42, 43]. This led to the establishment of the endometrial receptivity array test, using microarray molecular analysis; however, this method also identified delays in 25% of endometrial biopsies [29].

Previous studies on patients with RIF have shown a strong skew toward down-regulation of the expression of endometrial receptivity genes [44, 45]. Given the findings of the present study, it may be speculated that the genes were not normally up-regulated in the endometrial samples of RIF patients reported by Koler et al. [44] and Bastu et al. [45], possibly due to delayed transformation processes in the endometrium at the time of biopsy [46]. To support this hypothesis, it has been reported that endometrial samples from patients with RIF show a lack of typical secretory changes in comparison with fertile patients; this indicates an early secretory stage of the endometrium in patients with RIF and does not imply that they are unable to achieve mature endometrium with a receptive window of implantation [47]. The present study, with special endometrial monitoring of secretory phases in one menstrual cycle, shows that the human endometrium in subfertile patients is able to enter into the receptive implantation window, but often with a delay of 2–3 days. It appears that the delay in endometrial transformation is not age related, since the finding was observed in all of the age groups included, from 26 to 46 years. In support of this, studies with large sample sizes have shown that there is no statistically significant correlation between the patients’ age and the incidence of various types of endometrial receptivity deficiency [48, 49].

Individual secretory transformation, with a mean delay of two or more days, combined with insufficient compensation during the mid-secretory phase, as in late-onset changes, may lead to implantation failure due to mismatching of embryo implantation and endometrial receptivity. In IVF/ICSI cycles, it is possible to overcome this problem by carrying out embryo transfers that take into consideration the endometrial transformation status during artificial cycles. The correct timing of embryo transfers should be obtained by taking into account the two-biopsy analysis result from a prior mock cycle, in order to synchronize the delayed endometrium with the developing embryo [50]. The endometrial biopsies required with this method do not appear to be harmful for implantation, since several studies have demonstrated significantly higher implantation rates in patients in whom endometrial scratching was carried out during a previous cycle or in the ongoing cycle [51–55].

The results of the present study cannot be adapted on a one-to-one basis for stimulated in vitro fertilization cycles, since ovarian stimulation alters the protein expression profile in endometrial secretion, as reviewed by Li and Jin [56]. For example, the expression of endocrine gland-derived vascular endothelial growth factor protein has been reported to be significantly lower in stimulated cycles with a high ovarian response in comparison with natural cycles [57].

In contrast to molecular analyses of the endometrium, this method of monitoring analysis is able to identify sampling errors in which the biopsy only contains endocervical tissue or an admixture of endocervical and endometrial tissues. With the present method, protein expression pattern can be evaluated cell specifically (e.g., stroma, epithelium, vessels, immunocompetent cells), whereas in molecular analysis, the mRNA expression of genes is evaluated in a mixture of all cell types. Moreover, the present method analyses the protein expression pattern, rather than mRNA, and it may therefore better reflect the actual state of endometrial maturation, since many mRNAs are not translated into functional proteins. These facts are able to influence molecular analyses and might lead to misinterpretation of the results. With the present method, it is also possible to identify hyperplasia and atypia in the tissue, which can take place after hormonal therapy in women over 40 years of age.

## Conclusion

This method of monitoring secretory phases disclosed, for the first time, that the endometrium has individual dynamic transformation speeds and can compensate for or extend an initial “delay,” which is therefore a normal individual transformation process during the secretory phase, rather than only a fixed delay. The individual transformation speed may be due to a pacemaker, with constant, delayed, and augmented alteration rates, resulting in personally varying opening and closing of the implantation window. The endometrium thus enters the window of implantation at different time points. Compensation for the late onset of individual transformation during the early and mid-luteal phases was observed, since more than 98% of the second biopsies at HRT + 10 and 100% of those at OV + 10 were within the receptive window of implantation. This information is, therefore, relevant for scientific investigations of dynamic changes in endometrial tissue, as well as for the timing of embryo transfers in clinical conditions.
